# Two putative glutamate decarboxylases of *Streptococcus pneumoniae* as possible antigens for the production of anti-GAD65 antibodies leading to type 1 diabetes mellitus

**DOI:** 10.1007/s10123-023-00364-y

**Published:** 2023-05-08

**Authors:** Ernesto García

**Affiliations:** Departamento de Biotecnología Microbiana y de Plantas, Centro de Investigaciones Biológicas Margarita Salas (CSIC), Ramiro de Maeztu 9, 28040 Madrid, Spain

**Keywords:** Type 1 diabetes mellitus, *Streptococcus pneumoniae*, Glutamate decarboxylases

## Abstract

Type 1 diabetes mellitus (T1DM) has been increasing in prevalence in the last decades and has become a global burden. Autoantibodies against human glutamate decarboxylase (GAD65) are among the first to be detected at the onset of T1DM. Diverse viruses have been proposed to be involved in the triggering of T1DM because of molecular mimicry, i.e., similarity between parts of some viral proteins and one or more epitopes of GAD65. However, the possibility that bacterial proteins might also be responsible for GAD65 mimicry has been seldom investigated. To date, many genomes of *Streptococcus pneumoniae* (the pneumococcus), a prominent human pathogen particularly prevalent among children and the elderly, have been sequenced. A dataset of more than 9000 pneumococcal genomes was mined and two different (albeit related) genes (*gadA* and *gadB*), presumably encoding two glutamate decarboxylases similar to GAD65, were found. The various *gadA*_Spn_ alleles were present only in serotype 3 pneumococci belonging to the global lineage GPSC83, although some homologs have also been discovered in two subspecies of *Streptococcus constellatus* (*pharyngis* and *viborgensis*), an isolate of the group B streptococci, and several strains of *Lactobacillus delbrueckii*. Besides, *gadB*_Spn_ alleles are present in > 10% of the isolates in our dataset and represent 16 GPSCs with 123 sequence types and 20 different serotypes. Sequence analyses indicated that *gadA*- and *gadB*-like genes have been mobilized among different bacteria either by prophage(s) or by integrative and conjugative element(s), respectively. Substantial similarities appear to exist between the putative pneumococcal glutamate decarboxylases and well-known epitopes of GAD65. In this sense, the use of broader pneumococcal conjugate vaccines such as PCV20 would prevent the majority of serotypes expressing those genes that might potentially contribute to T1DM. These results deserve upcoming studies on the possible involvement of *S. pneumoniae* in the etiopathogenesis and clinical onset of T1DM.

**Supplementary Information:**

The online version contains supplementary material available at 10.1007/s10123-023-00364-y.

## Introduction

Many different studies have shown that infectious agents can cause chronic illnesses and have illustrated the important role of the human microbiome in health and disease (Gargano and Hughes 2014). A balanced microbial ecosystem within the human body is termed “eubiosis” with a dominance of diverse beneficial microorganisms living in mutual harmony. “Dysbiosis” occurs when the usual microbial species presence and quantities are distorted. In general, dysbiosis is characterized by low microbial diversity and prevalence of pathogenic bacteria. Most studies showing the involvement of dysbiosis in a variety of human diseases have been performed mainly with the gut and/or oral microbiomes (see Suárez et al. 2020; Abdelbary et al. 2022; Afzaal et al. 2022for recent reviews). Particularly, gut dysbiosis is thought to be linked to various human diseases, such as type 1 diabetes mellitus (T1DM), cardiovascular diseases, obesity, inflammatory bowel disease, or cancer, although the mechanisms over gut microbiota exerting its positive or harmful impacts remain largely unclear (Afzaal et al. 2022; Majumdar et al. 2022).

T1DM is one of the most common chronic diseases in childhood and the major type of diabetes in a pediatric population, but can occur at any age (Katsarou et al. 2017; DiMeglio et al. 2018). T1DM is preceded by T cell–mediated destruction of insulin-producing β cells in pancreatic islets. CD8^+^ T lymphocytes are the most common immune cells within insulitic lesions, with CD4^+^ T cells present in lower numbers. By 2021, the global estimated number of children and adolescents (0–19 years) with T1DM was 1.2 million, while the number of newly diagnosed cases each year was around 184,000 (International Diabetes Federation 2021). India has the highest estimated number of prevalent (existing) T1DM cases in people under 20 years of age (229,400), followed by the USA (157,900) and Brazil (92,300), whereas Finland ranked first when incidence rates (new cases) are considered (≈ 52 per 100,000 population per year) (International Diabetes Federation 2021). T1DM is a complex condition in which expected good outcomes can only be achieved with multiple daily insulin injections or the use of a controlled-release pump for insulin administration. Of interest, newly diagnosed and established T1DM patients are at increased risk of hospitalization for infectious diseases with a longer admission (Piccolo et al. 2022).

The causes of the destructive process of β cells in the pancreas are not fully understood, but a likely explanation is that the combination of genetic susceptibility (conferred by a large number of genes) (Redondo et al. 1999) and an environmental trigger—such as gut dysbiosis including changes to the gut microbiota, intestinal permeability, and intestinal inflammation—can initiate the autoimmune reaction (Vatanen et al. 2018; Dedrick et al. 2020; Geravandi et al. 2020; Garabatos and Santamaria 2022; Houeiss et al. 2022; Majumdar et al. 2022; Zajec et al. 2022; Zorena et al. 2022). A possible infectious origin (in this particular case, the mumps virus) for T1DM was already proposed nearly a century ago (Gundersen 1927). To date, many studies have dealt with the possible involvement of different enteroviruses (more specifically, coxsackievirus) in T1DM (for two recent comprehensive reviews, see Isaacs et al. 2021 and Lloyd et al. 2022). Another factor supporting an infectious basis for T1DM is seasonality (Gamble et al. 1969). Different studies have reported that there is a seasonal variation in the incidence of T1DM with a peak in the fall and winter months (Gerasimidi Vazeou et al. 2016 and references therein). Islet autoimmunity is serologically confirmed by the presence of at least one of the major T1DM-related autoantibodies (AAbs) in humans, i.e., insulin AAbs, glutamate decarboxylase 65 (65 kDa isoform) (designated as GAD65) AAbs, protein tyrosine phosphatase islet antigen 2 AAbS, and zinc transporter 8 AAbS (So et al. 2021). Glutamate decarboxylase (EC 4.1.1.15), a member of the group II pyridoxal-5′-phosphate (PLP)-dependent decarboxylases, catalyzes the irreversible α-decarboxylation of L-glutamic acid to γ-aminobutyric acid (GABA) and CO_2_, consuming one intracellular proton in the reaction. This enzyme is widely distributed in eukaryotes as well as prokaryotes (Ueno 2000), where it and GABA fulfil different physiological functions. GABA is the major inhibitory neurotransmitter in the mammalian central nervous system where it regulates neuronal excitability playing a key role in anxiety and depression disorders (Cryan and Kaupmann 2005), and has various physiological functions, including antioxidant, hypolipidemic, anti-inflammatory, and diuretic effects (Yogeswara et al. 2020). Outside of the central nervous system, high concentrations of GABA (≈ 20 mmol/g) are found within the insulin-producing β cells of the human pancreas distributed between endoplasmic reticulum/Golgi membrane-anchored, vesicular, and cytosolic localizations.

Molecular mimicry (MM) is the term used to describe sharing of antigenic determinants between the host and a parasite (Damian 1964). Bacterial “eukaryotic-like” proteins are a notable example of MM as they strongly resemble proteins (or carry domains) that are predominantly present in eukaryotes but are generally absent from prokaryotes. One of the best-studied cases of MM is that of acute rheumatic fever resulting from the immune response against *Streptococcus pyogenes* (group A) infection (Cunningham 2019). In relation with T1DM, Kaufman and coworkers (1992) first reported that the nonstructural protein P2C of coxsackievirus B, which appears to form part of the membrane-bound replication complex, shared a remarkable sequence similarity—an identical six-amino-acid sequence (PEVKEK) and several other conserved substitutions—with GAD65 and proposed that this is a mechanism by which this virus triggers T1DM. It has also been reported that other peptide sequences of the P2C protein of coxsackievirus B show a high binding affinity for several human leukocyte antigen (HLA) class II molecules, namely, HLA-DR4, -DR3, -DQ2, and -DQ8, and, therefore, they could also be involved in T1DM development (Ellis et al. 2005). Besides, the heat-shock protein 65 of *Mycobacterium avium* subsp. *paratuberculosis* (Hsp65_MAP_) and of *Mycobacterium tuberculosis* (Hsp65_Mtb_) also share great similarity with the pancreatic GAD65 in an antigenic peptide region (QERLAKLAGGVAVIKA) (Naser et al. 2013; Ozana et al. 2022). Amino acid sequence similarity between a putative *Streptococcus pneumoniae* (the pneumococcus) glutamate decarboxylase A (GadA_Spn_) and GAD65 has also been reported (García and López 1995). In that study, only serotype 3 pneumococci were found to harbor a *gadA*_Spn_ gene; unfortunately, the lack of genomic data precluded any further investigation at that time. Recently, an analysis of the similarities between different bacterial glutamate decarboxylases and GAD65 has been published (Bedi et al. 2022).

In the present study, strong evidence showing that *gadA*_Spn_ alleles are restricted indeed to serotype 3 pneumococci (and some other related bacterial species) is provided. In addition, a novel gene (*gadB*_Spn_) closely related with *gadA*_Spn_ (≈ 50% sequence identity) has also been found in a variety of pneumococcal isolates of different serotypes and sequence types (STs). The implications of these findings in the context of a possible role of pneumococcus in the triggering of autoimmunity in T1DM onset and development are discussed.

## Materials and methods

### Compilation of the pneumococcal genome dataset and bioinformatic analyses

The National Center for Biotechnology Information (NCBI) database (available at https://www.ncbi.nlm.nih.gov/genome/?term=Streptococcus+pneumoniae) (last accessed, November 3, 2022) includes 9055 whole genome sequences of *S. pneumoniae* (assembled or otherwise) that were carefully mined. Multilocus sequence typing (MLST) is a standard tool in population genetics and bacterial epidemiology that assesses the genetic variation present in a reduced number of housekeeping genes (typically seven) along the genome (Enright and Spratt 1998). When not reported, sequence types (STs) and clonal complexes (CCs) were determined on the basis of whole genome sequencing data (Jolley et al. 2018). When needed, single (SLVs) and double locus variants (DLVs) were also annotated. In some cases, sequence-based methods were used to ensure accurate serotype prediction (van Tonder et al. 2016). Whenever possible, the isolate descriptions included the lineages defined as Global Pneumococcal Sequence Clusters (GPSCs) (Gladstone et al. 2019).

Sequence comparison and alignments were performed using the BLAST platform and/or Clustal Omega package (Sievers and Higgins 2021) running at the European Bioinformatics Institute (EMBL-EBI) website. When required, the mol % G + C content was determined using the GC Content Calculator running at the VectorBuilder Inc. website (https://en.vectorbuilder.com/tool/gc-content-calculator.html).

The AlphaFold Protein Structure Database (AlphaFold DB, https://alphafold.ebi.ac.uk) was used to make structure predictions of GadA_Spn_ (Q59956_STREE) and GadB_Spn_ (A0A0T8EX30_STREE) (Jumper et al. 2021; Varadi et al. 2022). The corresponding files were visualized using FirstGlance in Jmol (Version 4.1) (https://proteopedia.org/wiki/fgij/). To reveal regions that can be important for the structure and*/*or function of a protein, an analysis of the evolutionary pattern of the amino acid residues was performed at the ConSurf web server (https://consurf.tau.ac.il) (Ashkenazy et al. 2016).

## Results

### Serotype 3 pneumococci belonging to GPSC83 harbor a *gadA*_Spn_ gene

At the present time, the dataset of *S. pneumoniae* isolates harboring a *gadA*_Spn_-like gene contains 40 strains, including two strains whose genome was sequenced to complete assembly, i.e., A66 (= NCTC 7978) and SPNA45 (Table S1). All these 40 strains were of serotype 3, in agreement with previous Southern blotting results with a low number of strains (2/11) (García and López 1995), and were isolated mainly from blood or cerebrospinal fluid. Based on ST, 38 out of the 40 strains belong to CC378 constituted by ST378 and its SLVs (ST232, ST1377, and ST7369) and DLVs (ST260, ST6014, ST11931, and ST16577). Two singletons (ST369 and ST6934) were also found. As a whole, the majority of the pneumococcal isolates (38/40) with a 1428 bp *gadA*_Spn_-like gene belong to GPSC83, a global pneumococcal sequence cluster of intermediate frequency (Gladstone et al. 2019). These authors included in GPSC83, 13 isolates of CC1220, 9 of CC378, and a single isolate of ST11931. Five isolates were ST1220 and eight were ST260 (a SLV of ST1220). Of note, all of the ST260 isolates harbored a copy of the *gadA*_Spn_ gene (Table S1) whereas those of ST1220 did not (WGS projects CABAFM01, FHPC02, CAANYB01, CAAPEX01, and CAAPYY01 [data not shown]). The genomic zones of these ST1220 isolates where the *gadA*_Spn_ gene should be located are 98% identical to the SPD_RS05895–SPD_RS05910 region of strain D39.

**Fig. 1:**
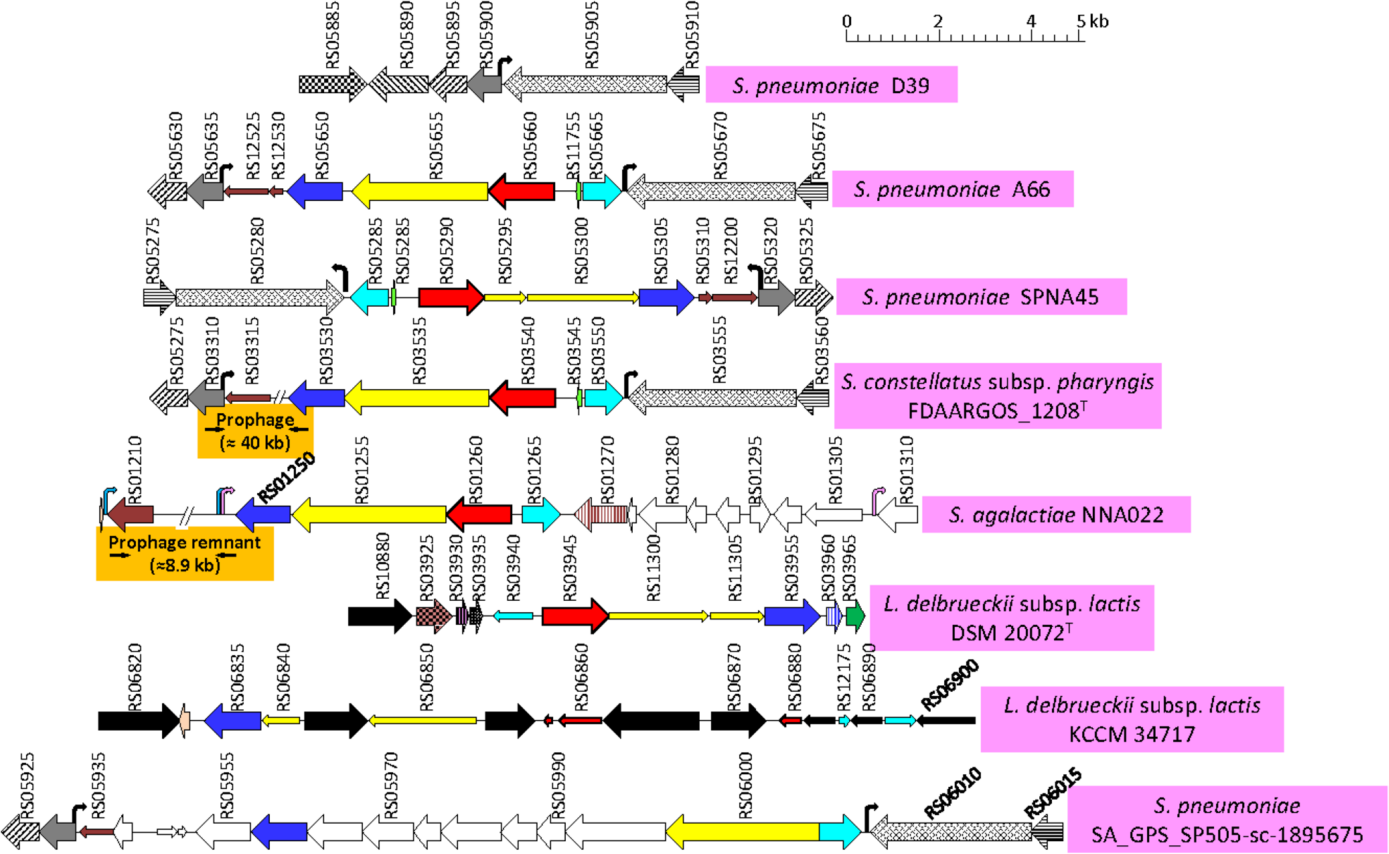
Schematic representation of the chromosomal region surrounding a *gadA*-like gene in several bacterial species. Genes are shown with arrows pointing in the direction of transcription. Homologous genes are represented by identical color and/or shading. Red arrows correspond to the *gadA*-like genes. Dark and light blue arrows represent genes putatively coding for a transporter of the major facilitator superfamily (MFS) and a transcriptional regulator of the Rgg/GadR/MutR family, respectively. Yellow arrows correspond to genes putatively encoding a nonribosomal peptide synthase. Thin arrows represent interrupted genes (pseudogenes). Insertion sequences are shown as black arrows. Open arrows represent genes that are irrelevant in this study. The corresponding genomic regions of *S. pneumoniae* D39 and SA_GPS_SP505_sc_1895675 (GPSC21 ST10619 serotype 19F; NZ_LR216035) are shown for comparison

Four different *gadA*_Spn_ alleles were found—allele 3 being the most frequent—that encodes a 475-amino acid protein; the most frequent GadA_Spn_ allele is included in the NCBI database under accession number WP_061632578 (Table S1). Since the four *gadA*_Spn_ alleles were ≥ 97.8% identical, allele 3 was chosen for further analyses, unless stated otherwise. When the genome of *S. pneumoniae* A66A was compared with that of strain D39 (NC_008533.2), it could be determined that the *gadA*_Spn_ gene (marked as a red arrow in Fig. 1; locus tag A66_RS05660) is included in a *ca*. 9 kb DNA fragment inserted at the 5’ end of SPD_RS05900, a locus potentially encoding a Cof-type HAD-IIB family hydrolase. In D39, the insertion site is 5’-1,134,639 TCCATATCCGTTGCTACTAGTTTAAT 1,134,664–3’, whereas in strain A66 (and other pneumococci) the inserted DNA fragment is flanked by a near-perfect, direct repeat: 5’-1,095,234 TCCATATCTGTTGCTACTAGTTTAAT 1,095,259–3’, and 5’-1,104,316 TCCGATTTCTGTTGCTACTAATTTAAT 1,104,342 -3’. The same situation was found in *S. pneumoniae* SPNA45, although the coding sequences were inverted, as compared with strain A66. This is due to the fact that this DNA region is located in one of the rearranged fragments of SPNA45 (Morales et al. 2015). In addition, the gene A66_RS05665, coding for a putative Rgg/GadR/MutR family transcriptional regulator, is 45% similar (25% identical; *E* = 8 × 10^−15^) to the *gadR* activator of the *Lactococcus lactis gadCB* operon (Sanders et al. 1998).

Sequence comparisons of *gadA*_Spn_ with those present in the databases revealed the presence of a very similar (98% identity) gene of identical length in *Streptococcus constellatus* (*gadA*_Sco_) and, specifically, in two phylogenetically closely related subspecies, i.e., subsp. *pharyngis* and subsp. *viborgensis* (Fig. 1 and Table S1) (Whiley et al. 1999; Jensen et al. 2013). In addition, all these *S. constellatus* strains share the same *gadA*_Sco_ allele. Furthermore, these genes are syntenic, this is, they share the same order of genes, and the DNA fragment where they are contained in *S. constellatus* is > 90% identical to that of *S. pneumoniae* A66, whereas the flanking genes were about 75% identical, as expected for two species of the same genus (Fig. S1A). This suggests that the *gadA*_Sco_ gene may have been recently (in evolutionary terms) incorporated into the genome of an ancestor of the two related subspecies of *S. constellatus* and that this integration probably involved a pneumococcal donor. Remarkably, at the position of loci A66_RS12525 and A66_RS12530 potentially encoded proteins involved in recombination, there exists a *ca*. 40 kb prophage both in *S. constellatus* subsp. *pharyngis* and *S. constellatus* subsp. *viborgensis*. This prophage is identical, over a 37,901 bp overlap, to the Javan113 prophage (Acc. No. MK448670) previously reported (Rezaei Javan et al. 2019) and very similar to prophages from other streptococcal species (Fig. S1B). Furthermore, other *gadA* genes also similar to that of *S. pneumoniae* were found in four strains of *Streptococcus agalactiae* (group B streptococci) and in the genome of 24 strains of *Lactobacillus delbrueckii*, mainly in *L. delbrueckii* subsp. *lactis* (22 strains) (Table S1). The nucleotide identity between *S. agalactiae gadA* (*gadA*_Sag_; 1434 bp) and *gadA*_Lde_ (1422 bp) from *L. delbrueckii* is *ca.* 65%, a value very close to the average sequence identity (68%) between these genes and those of *S. pneumoniae* (or *S. constellatus*) (not shown). The most divergent region of the gene is located in its 5’ part (between nucleotide positions 1 and 200, approximately). Of note, 6 out of 22 strains of *L. delbrueckii* subsp. *lactis* contain an incomplete copy of the *gadA*_Lde_ gene (Table S1). Interestingly, *L. delbrueckii* subsp. *lactis* KCCM 34,717 contains many insertion sequences (ISs) that interrupt *gadA*_Lde_ and several other flanking genes. This finding was not completely unexpected since it has been proposed that ISs play an important role in the evolution of *Lactobacillus* species (Kaleta et al. 2010). A diagram showing the chromosomal region containing a *gadA*-like gene in different bacterial species is shown in Fig. 1.

It is interesting to point out that, at least, four additional pneumococcal genomes harbor a different (but partly related) insert of about 17 kb, at the same position where the 9 kb insert containing the *gadA*_Spn_ gene is located. These are SA_GPS_SP505-sc-1895675 (GPSC21 ST10619 serotype 19F; NZ_LR216035) (Fig. 1), 2245STDY5775874 (GPSC90 ST8328 serotype 19F; NZ_LR216032), 2245STDY5699394 (GPSC30 ST7055 serotype 10B; NZ_LR216024), and B1900 (serotype 3; NZ_CP051650). In these cases, as in those containing *gadA*_Spn_ (see above), the insert is flanked by a near identical repeated sequence, e.g., 5’-1,107,742 TCCATATCCGTTGCTACTAGTTTAAT 1,107,767–3’ and 5’-1,124,932 TCCAATTTCTGTCGCTACTAGTTTAAT 1,124,958–3’ in the particular case of strain SA_GPS_SP505-sc-1895675. It should be underlined that the pneumococcal *smc* gene (corresponding to SPD_RS05905 in D39 and represented by cross-hatched arrows in Fig. 1), has been identified as a hotspot for both recent and ancestral recombination events (Mostowy et al. 2017). The *smc* gene encodes the condensin protein SMC and is not essential in *S. pneumoniae*—albeit it is important for timely localization of the division site (van Raaphorst et al. 2017). A similar role for *smc* has recently been demonstrated in *S. agalactiae* (Lee and Andam 2022).

**Fig. 2:**
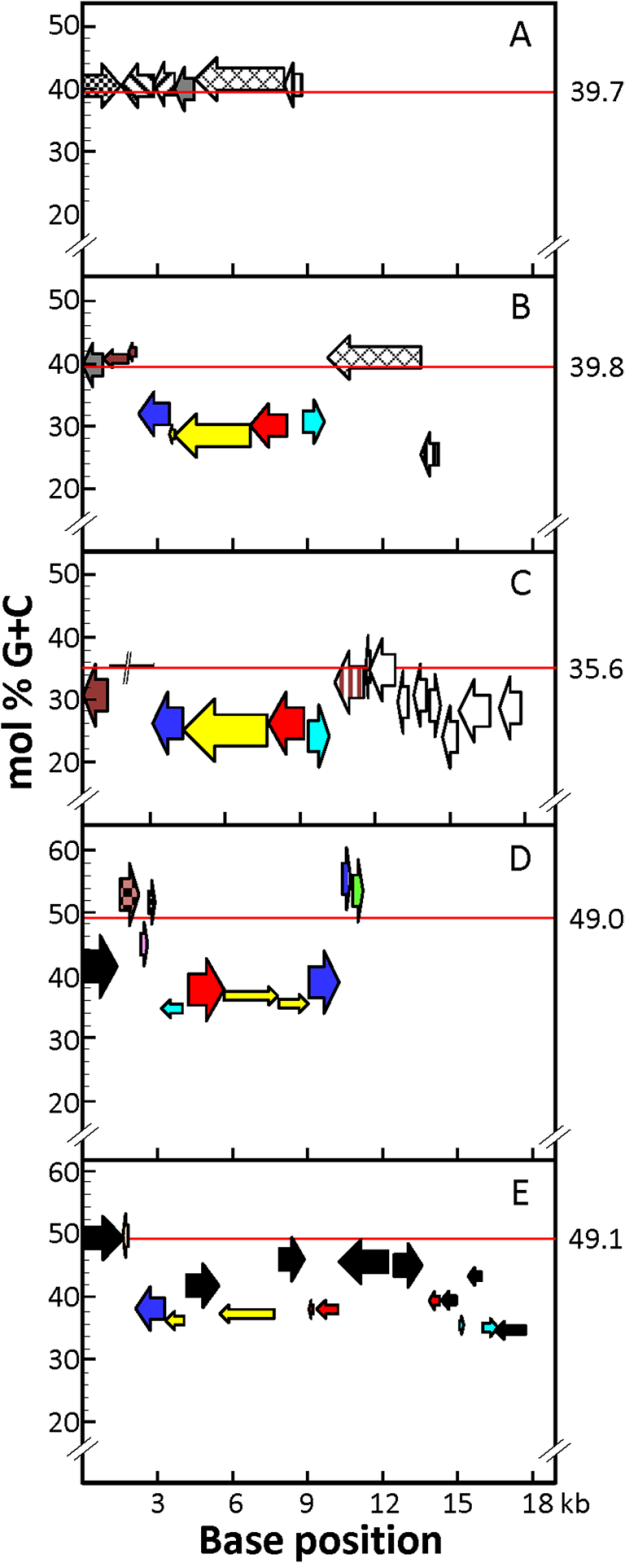
Mol % G + C content of genes included in the DNA region encompassing *gadA*-like genes. The G + C content of each chromosome is indicated by a red line and at the right of the figure. Panels A to E correspond respectively to *S. pneumoniae* D39, *S. pneumoniae* A66, *S. agalactiae* NNA022, *L. delbrueckii* subsp. *lactis* DSM 20072^ T^, and *L. delbrueckii* subsp. *lactis* KCCM 34717. The color code of genes is the same as in Fig. 1. The mol % G + C content was calculated for each gene

Another interesting feature of the *gadA*_Spn_-containing fragment is the low mol % G + C content (≈ 30%), much lower than that of *Streptococcus* species (35–40%) or *L. delbrueckii* (≈ 50%) (Fig. 2). Foreign DNA incorporated into a genome may have a different G + C composition. Over time, such DNA is subjected to a process of amelioration where directional mutation pressures act to alter the base composition of the incoming DNA to match that of the whole genome (Bentley and Parkhill 2004).

### A novel gene (*gadB*_Spn_) is present in a diversity of *S. pneumoniae* strains

**Fig. 3:**
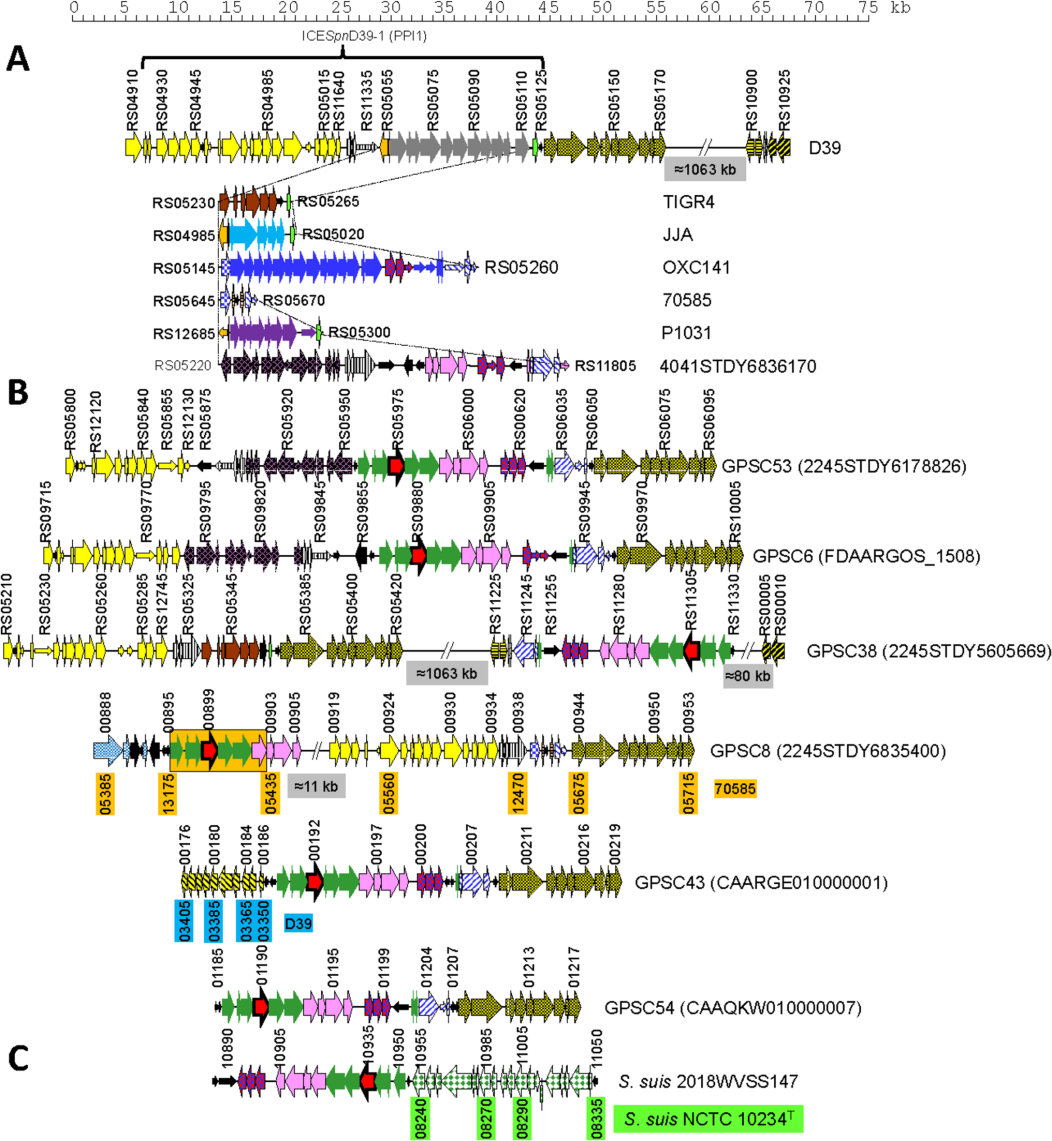
Diagram showing the gene organization around the *gadB* gene in selected *S. pneumoniae* strains. **A** Gene content of the variable region located between SPD_RS11335 and SPD_RS05125 of strain D39 in other pneumococcal genomes. **B** The same region as that showed in panel A but in strains harboring a *gadB*_Spn_ gene (indicated by a red arrow). The cluster of seven genes that are conserved in all of the pneumococcal genomes studied is highlighted in an orange rectangle. The nucleotide sequence of the genome of the GPSC9 strain (CAAYNA010000008) at this region (between positions 28,233 and 93,432) is > 99% identical to that shown for strain FDAARGOS_1508 (GPSC8) (1,869,701–1,935,072). **C** The corresponding region in the genome of a *S. suis* isolate. In panels **B** and **C**, the gene organization of *S. pneumoniae* 70585 (NC_012468) and *S. suis* NCTC 10234^ T^ (NZ_LS483418.1), respectively, is also shown for comparison

In addition to identifying pneumococcal isolates harboring a *gadA*_Spn_ gene, sequence comparisons revealed the existence of another gene (designated as *gadB*_Spn_ hereafter) in 1591 strains whose genomes were available from the NCBI database (Table S2). From these, 1182 isolates could be assigned to 16 different GPSCs and represent 123 STs and 20 serotypes (Table 1). The vast majority (92.4%) of isolates belong to GPSC6 (424), GPSC8 (363), GPSC38 (110), GPSC53 (100), and GPSC43 (95). Notably, only one member (BioSample: SAMEA3233202) of GPSC9—a dominant cluster—harbored a *gadB*_Spn_ gene. Pairwise sequence alignments showed that GadA_Spn_ (475 amino acid residues) and GadB_Spn_ (501 amino acid residues) contain 37% identical amino acid residues and 58% conserved substitutions (Table 2). In an analogous way to that already mentioned for *gadA*_Spn_, the *gadB*_Spn_ gene was found to reside in a DNA fragment together with a variety of other genes (Fig. 3).

**Table 1:** Distribution of STs and serotypes among GPSCs harboring *gadB*_Spn_

GPSC	Prevalence^a^	*n*^b^	Different STs	Most frequent STs (*n*)	Serotypes^c^	Most frequent serotypes (*n*)
6	Dominant	424	49	156 (276); 162 (47); 143 (19); 1269 (9)	10	14 (242); 9 V (139); 19A (22); 15B/C (10); 11A (4)
8	Dominant	363	10	289 (232); 5659 (47); 3404 (33); 7050 (28)	1	5 (363)
9	Dominant	1	1	861 (1)	1	14 (1)
38	Intermediate	110	10	393 (78); 310 (16); 9325 (6); 5475 (4)	1	38 (110)
43	Dominant	95	17	280 (31); 3214 (21); 239 (18); 11,758 (10)	7	9 V (65); 35A (23)
53	Intermediate	100	5	847 (90); 5262 (5); 11,714 (3)	1	19A (100)
54	Intermediate	32	7	9473 (13); 5778 (7); 6317 (5); 706 (3)	2	9 V (30)
155	Intermediate	13	5	105 (5); 5604 (5)	1	25F (13)
172	Rare	16	4	6693 (13)	2	20 (15)
208	Intermediate	10	2	4908 (8)	1	9 V (10)
234	Rare	7	4	10,606 (3); 1116 (2)	1	3 (7)
247	Rare	3	3	613 (1); 7616 (1); 12,796 (1)	–	NT (3)
257	Rare	5	3	5407 (3)	2	25F (3); 38 (2)
419	Rare	1	1	6346 (1)	1	18C (1)
536	Rare	1	1	5359 (1)	1	38 (1)
566	Rare	1	1	4651 (1)	1	18F (1)
Total		1182	123		20	

^a^The data of prevalence of the indicated GPSC lineages were taken from Gladstone et al. (2019)

^b^Number of isolates

^c^Due to repetitions of some serotypes in various lineages, the total number of different serotypes is 20

**Table 2:** Pairwise comparisons between different GadA and GadB proteins^a^

	GadA_Sco_	GadA_Sag_	GadA_Lde_	GadB_Spn_	GadB_Ssu_
GadA_Spn_	97/98	64/80	67/82	37/58	37/57
GadA_Sco_		64/80	68/83	37/58	37/58
GadA_Sag_			62/80	38/57	38/57
GadA_Lde_				35/55	35/55
GadB_Spn_					99/100

^a^Numbers indicate the percentage of identical/conserved amino acid residues. *Spn*, *S. pneumoniae*; *Sco*, *S constellatus*; *Sag*, *S. agalactiae*; *Lde*, *L. delbrueckii*; *Ssu*, *S. suis*

Sequence alignments indicated that the insertion of foreign genes (including *gadB*_Spn_) may result in the insertion and/or deletion of a variable number of the genes of the recipient strain. In most cases, the acquisition of *gadB*_Spn_ involves a region that, in the D39 strain, is located between the termination codon of SPD_RS04910 (*rlmD* = *rumA*) and the initiation codon of SPD_RS05130 (37.5 kb) (Fig. 3A). A recent study has identified this region as ICE*Spn*D39-1 (Liu et al. 2019). Integrative and conjugative elements (ICEs) are mobile genetic elements (MGEs) integrated into bacterial genomes, which encode their own excision, conjugative transfer, and integration (Haudiquet et al. 2022). Previously, this area had been named PPI1 (for Pneumococcal Pathogenicity Island 1), and includes four genes (*piuBCDA*; from SPD_RS04930 to SPD_RS04945 in the D39 genome) coding for proteins involved in iron transport by *S. pneumoniae* (Brown et al. 2001, 2002); at least one of the other genes in this region (SPD_RS05200 in D39 or SP_1051 in TIGR4; NC_003028.3) has been reported to contribute to virulence in a mouse model of infection (Brown et al. 2004). More recently, it has been demonstrated that SPD_RS05195 (coding for PezA) and SPD_RS05200 (encoding PezT) actually correspond to the pneumococcal epsilon-zeta homolog (PezAT)—a class II, functional toxin–antitoxin (TA) system—with PezA as the cognate antitoxin to the PezT toxin (Khoo et al. 2007). PPI1 is a putative mobile variable region (Wyres et al. 2013) present in highly virulent isolates but not in non-invasive or intermediate-virulent strains (Mutschler et al. 2011). It has also been reported that *pezAT* mutants exhibit higher resistance to β-lactam antibiotics and enhanced genetic competence (Chan and Espinosa 2016). As already mentioned, this zone greatly varies among different *S. pneumoniae* isolates, not only in size (changing to up to ≈ 58 kb in strain 70585), but also in gene composition. Interestingly, similarity searches showed that the proposed ICE of strain 70585 contains genes very similar (77–93% nucleotide identity) to 27 ORFs (ORF1–16, ORF69, ORF75–80, and ORF86) out of the 86 predicted genes of the 94-kb ICE*Slu*van element of *Streptococcus lutetiensis*, which also inserts at the 3’ end of *rlmD* (Bjørkeng et al. 2013). The finding that different pneumococcal strains contain dissimilar gene cassettes (Fig. 3A) is in agreement with a role as a recombination hotspot. Indeed, the variability degree of this zone is not strain-specific and some strains share a near-identical syntenic organization. The great diversity of genes accompanying *gadB*_Spn_ is depicted in Fig. 3B. This gene (indicated by a red arrow) is encompassed by genes that may vary in number, orientation, and function depending on the pneumococcal strain analyzed. Many similarities, however, exist in this region; for example, between strains 224STDY6178826 (GPSC53 ST947; NZ_LR216061) and FDAARGOS_1508 (GPSC6 ST156; NZ_CP083627), synteny is very obvious, except for a group of genes that are arranged in opposite directions (indicated in Fig. 3B as black arrows cross-hatched with yellow lines).

In addition to the insertion site indicated above (between SPD_RS04910 and the initiation codon of SPD_RS05130, taking the D39 genome as a model), a second insertion site exists between SPD_RS10915 and SPD_RS10920. This region is located far away from the previous one (about 1 Mb apart) (Fig. 3A). This appears to be the case of strain 2245STDY5605669 (GPSC38 ST310; NZ_LR216017), although the genes E0F14_RS11240 (homolog of SPD_RS10915) and E0F14_RS00005 (corresponding to SPD_RS10920 in D39) were separated by ≈ 80 kb, instead of being contiguous, as is the case in D39. On the other hand, in strain 2245STDY6835400 (GPSC8; CAAVMP010000004), the *gadB*_Spn_ gene is located ≈ 11 kb upstream of the cluster of genes located 3’ of SPD_RS05055 (in strain D39). When compared with strain 70585, the location of *gadB*_Spn_ in strain 2245STDY6835400 was found to be flanked by SAMEA104035315_00895—encoding a frameshifted IS*5*-like element and corresponding to SP70585_RS13175 in strain 70585—and SAMEA104035315_00903, which matches to SP70585_RS05435 and encodes a putative chloramphenicol acetyltransferase (CAT) (Fig. 3B). Immediately downstream of the CAT-coding gene, two more genes putatively encoding, respectively, a methionine–tRNA ligase (SAMEA104035315_00904) and a tyrosine-protein phosphatase (SAMEA104035315_00905), were found in every pneumococcal strain harboring a *gadB*-like gene. A detailed analysis of the genes shown in Fig. 3B revealed that the minimum cassette embracing *gadB*_Spn_ appears to be composed of seven genes encoding respectively: (1) a hypothetical protein (HP), (2) an acyl carrier protein, (3) another HP, (4) the glutamate decarboxylase GadB_Spn_ itself, (5) an acyl–CoA ligase, (6) an aminotransferase class I/II-fold pyridoxal phosphate-dependent enzyme, and (7) another HP. These genes are indicated by light green and pink arrows in Fig. 3B and included in an orange rectangle, and their mol % G + C content ranges between 27.0 and 31.9. In most strains, however, this minimum cassette is accompanied by three more genes in position 3’ of the last of the seven genes and those putatively encoding CAT, methionine–tRNA ligase, and the tyrosine-protein phosphatase already mentioned (also indicated by pink arrows). All these genes have a mol % G + C content lower than that of the whole chromosome (Fig. S2). In four out of six strains, the gene cassette including *gadB*_Spn_ is located ≈ 8 kb upstream of a group of genes designated as SPD_RS05125 to SPD_RS05170 in strain D39 (Fig. 3B).

As mentioned above, *gadA*_Spn_ homologs are present in several Gram-positive bacteria; these are *S. constellatus* subsp. *pharyngis*, *S. constellatus* subsp. *viborgensis*, *S. agalactiae*, and in three subspecies of *L. delbrueckii*. Remarkably, a *gadB*-like gene was found in two isolates of *Streptococcus suis* (Fig. 3C), which presumably correspond to a single strain: the two isolates (2018WUSS147 and 2018WUSS150) were sampled in the same day (August 27, 2018) and in the same city (Hunan, China), and identified at the same laboratory (OIE Reference Laboratory for Swine Streptococcus). Even more, there exists near 100% nucleotide identity between the contigs of the corresponding *S. suis* isolates (unpublished observations). Interestingly, the pneumococcal allele 1 and the swine alleles of *gadB* are closely related since they are of the same length (1506 bp), they differ only at two nucleotide positions, and their encoded proteins differ only by a conserved, single amino acid substitution (Met in GadB_Spn_ → Ile486 in GadB_Ssu_).

In addition to the case of *S. suis*, *gadB*_Spn_*-*like genes appear to exist in some Gram-positive strains, mainly in some members of the *Bacillus* genus. The most similar homolog of GadB_Spn_ (56% identity, 75% similarity) is encoded by the WR52_RS29730 locus of the *Bacillus cereus* (strain HN001) plasmid pRML02 (Fig. S3). The GadB_Bce_ decarboxylase has 504 amino acid residues, similar to the 501 amino acid residues of GadB_Spn_. Notably, several of the genes located around *gadB*_Spn_ are preserved around *gadB*_Bce_, although the synteny is somehow different (Fig. S3). Another protein very similar (> 98% identity) to GadB_Bce_ is WP_097888410 (504 amino acid residues; GadB_Bth_) that is encoded by, at least, six different strains of *Bacillus thuringiensis* (Table S2).

### GadA and GadB have putative epitopes similar to those of GAD65 presumably involved in T1DM development

Although many vertebrates harbor 3 different GAD-coding genes (Grone and Maruska 2016), GAD exists in two isoforms in humans, GAD67 and GAD65, each encoded by a different gene, *GAD1* and *GAD2*—located in chromosomes 2 and 10, respectively—which differ in size, charge, localization, and antigenicity (Erlander et al. 1991; Kassa et al. 2014). GAD67 exists as the active holoenzyme (bound to PLP) that provides a steady production of neuronal cytosolic GABA, whereas GAD65 predominantly exists as a PLP-dissociated apoenzyme that mediates activity-dependent GABA synthesis when fast postsynaptic inhibition is needed switching from the inactive to the active form. GAD65 AAbs (but not GAD67 AAbs) were detected in 80–90% of newly diagnosed patients and were an early marker of β cell destruction in individuals who later developed disease (Atkinson et al. 1990). GAD67 isoform AAbs have been detected in the serum and the cerebrospinal fluid of patients with various neurological syndromes, although those AAbs are barely detected in the absence of GAD65 AAbs and thus are not considered clinically relevant.

According to its linear sequence, GAD65 is divided into three functional domains: the N-terminal domain comprising residues 1–188, the PLP domain (residues 189–464), and the C-terminal domain comprising residues 465–585 (Fenalti and Buckle 2010). The major epitopes in T1DM have been mapped to the PLP and C-terminal domains (Schwartz et al. 1999), and the elimination of the first 100 amino acid residues altered neither enzyme activity nor reactivity with sera from diabetic patients (Fenalti et al. 2007). In addition, AAbs to N-terminally truncated GAD65 (lacking the 95 N-terminal amino acids) have been reported to identify more specifically at-risk relatives of patients with T1DM than AAbs to full-length GAD65 (Pöllänen et al. 2022). In contrast, isolated positivity for AAbs to the N-terminal epitope of GAD65 confers no increased risk for T1DM. Pairwise sequence alignments of GAD65, GadA_Spn_, and GadB_Spn_ were performed (not shown) and the CD4^+^ and CD8^+^ epitopes compiled in previous publications (James et al. 2020; Amdare et al. 2021; Ivanov et al. 2022) were localized in the alignments (Table 3). Seven putative epitopes in the pneumococcal Gads were located at regions corresponding to positions 202–266 in GAD65 and three at its C-terminal domain. This fits with the observation that the PLP domain of GAD65 is the most immunodominant region both at diagnosis and thereafter (Ronkainen et al. 2004).

**Table 3:**
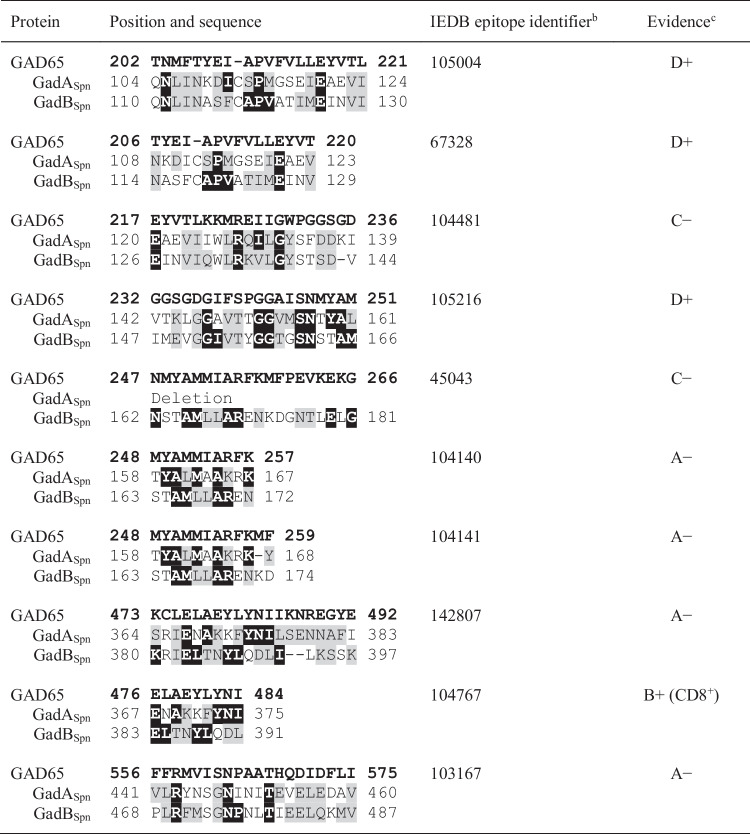
Sequence similarities between various GAD65 epitopes known to be relevant in T1DM and GadA_Spn_/GadB_Spn_^a^

^a^Residues on black or grey boxes indicate amino acids identical or conserved substitutions, respectively, between GAD65 and any of the two pneumococcal Gads. The sequences of T-cell epitopes were taken from James et al. (2020) and Amdare et al. (2021). ^b^IEDB: the Immune Epitope Database (https://www.iedb.org/home_v3.php). ^c^With the exception of 476 ELAEYLNI 484, the rest are CD4^+^ T-cell epitopes. The level of evidence that defines a given epitope as such was taken from James et al. (2020). Evidence of natural processing and presentation is not available for epitopes marked as C or D

Folding predictions of GadA_Spn_ and GadB_Spn_ using AlphaFold together with an evolutionary analysis of these proteins were done in a subset of the potential epitopes shown in Table 3, namely, peptides 158–167 and 367–375 of GadA_Spn_ (Fig. S4), and 163–172 and 383–391 of GadB_Spn_ (Fig. S5). The models indicated that, with the possible partial exception of peptide 163–172 (GadB_Spn_) that is somehow buried, the putative epitopes analyzed are located on the surface of the proteins, in well-conserved regions, and may be favored due to increased antibody accessibility.

## Discussion

There exists a complex interaction between genetic susceptibility, immune dysregulation, and environmental factors in the etiology and pathogenesis across all stages of T1DM. Over 50 regions of the human genome contain genes that have been identified as associated with T1DM susceptibility. Genes that confer the highest susceptibility are class II HLA genes (Robertson and Rich 2018). The “Environmental Determinants of Diabetes in the Young” (TEDDY) study (Vatanen et al. 2018) and other human longitudinal T1DM studies have evaluated environmental putative triggers, including infections and antibiotic use, birth mode, diet, and gut microbiota/virome (Dedrick et al. 2020). Potential environmental factors influencing the risk of T1DM have been identified although, for many of these issues, the evidence is low and often controversial still requiring proofs to support the pathways from seroconversion to β cell failure. MM has been repeatedly proposed as a possible mechanism of T1DM triggering. In addition to the already mentioned role of GAD65 autoimmunity, a recent study has reported that a 15 amino acid peptide from the Gram-negative anaerobic bacterium *Parabacteroides distasonis*, a normal human gut commensal, mimics an epitope in the B-chain of insulin and behaves as a molecular trigger of T1DM pathogenesis (Girdhar et al. 2022). Nevertheless, further studies are required since there are also pieces of evidence suggesting that *P. distasonis* may have both beneficial and detrimental effects on T1DM pathogenesis (Ezeji et al. 2021).

*S. pneumoniae* is a leading human pathogen, a major cause of non-invasive diseases such as acute otitis media (AOM), sinusitis, and conjunctivitis, and the main etiologic agent of community-acquired, bacterial pneumonia and meningitis. The pneumococcus is an encapsulated bacterium that can express more than 100 antigenically and biochemically distinct serotypes. Pneumococci colonize the upper respiratory tract (mainly the nasopharynx) where they may persist as a commensal (carrier state). Once the carriage is established, the pneumococcus may invade several sterile sites leading to what is known as an invasive pneumococcal disease (IPD) (Vernatter and Pirofski 2013). Recent data estimate that *S. pneumoniae* was the world leading cause of fatal lower respiratory infections with > 650,000 deaths in 2019 and that, among children younger than 5 years, it was the pathogen associated with the most deaths (GBD 2019 Antimicrobial Resistance Collaborators 2022). It has been estimated that, in 2004, the direct medical costs for pneumococcal diseases in the USA totalized $3.5 billion. Pneumonia (866,000 cases) accounted for 22% of all cases and 72% of pneumococcal costs. AOM and sinusitis (1.5 million cases each) comprised 75% of cases and 16% of direct medical costs (Huang et al. 2011). It is recognized that, in non-tropical climates, IPD displays wintertime predominance (Watad et al. 2017; Domenech de Cellès et al. 2019; Berry et al. 2020). Pneumococci usually associate with some seasonal respiratory viruses such as respiratory syncytial virus and influenza A virus, which have also been suggested to induce T1DM (Klugman et al. 2009; McCullers 2014; Thomas et al. 2022). Some recent studies have suggested that the COVID-19 pandemic was associated with increased diabetes risk (Barrett et al. 2022; Ssentongo et al. 2022). Interestingly, two recent studies have shown that pneumococcal carriage detection and density appear to be related with SARS-CoV-2 infection, suggesting a synergistic relationship in the upper airway (Mitsi et al. 2022; Parker et al. 2023).

In the present study, we have confirmed and extended a preliminary report on the presence of a GAD65-coding gene (*gadA*_Spn_) in a few serotype 3 *S. pneumoniae* isolates (García and López 1995). Now, we have confirmed that only serotype 3 strains harbor *gadA*_Spn_ and that 38 out of the 40 isolates analyzed here belong to GPSC83, more precisely to CC378 (including SLVs and DLVs) (Table S1). Remarkably, only ST1220 isolates of the GPSC83 lineage completely lacked *gadA*_Spn_. Serotype 3 strains form highly mucoid colonies and its capsular polysaccharide protects the bacterium from opsonophagocytosis (Neeleman et al. 1999). Serotype 3 is included in the polysaccharide 23-valent (PPSV23) vaccine as well as in the 13-valent pneumococcal conjugate vaccine (PCV13), 15-valent PCV (PCV15), and 20-valent PCV (PCV20). PCV15 and PCV20 contain additional serotypes to PCV13; PCV15 is already approved and commercialized for adults (aged 18 years and older) and for children (aged 6 weeks through 17 years), whereas PCV20 is used in adults and it would be promptly approved for children. Moreover, a novel 21-valent conjugate vaccine (V116) is currently under clinical evaluation (Platt et al. 2023). Following the introduction of PCVs in Europe and the USA, a reduction in pneumococcal AOM and the rate of IPD has occurred due to effectiveness against strains expressing serotypes in the PCVs implemented. However, IPDs and AOMs replaced by non-vaccine serotypes including those associated to antibiotic resistance have emerged and now predominate in high-income countries (de Miguel et al. 2021; Sempere et al. 2022; Pichichero et al. 2023). To date, *S. pneumoniae* still remains a major cause of serious human disease not only in the elderly (de Miguel et al. 2021), but also in the pediatric population where it associates with complicated pneumonia cases, often with empyema or pleural effusion (Silva-Costa et al. 2022). Moreover, recent results have demonstrated that serotype 3 pneumococci may cause adverse cardiovascular events during IPD (Africano et al. 2021). It has been noted that PCV13 did not elicit enough protective anti-serotype 3 Abs; one possible explanation for the failure of current vaccines against serotype 3 may be the release of capsular polysaccharide (Luck et al. 2020), but alternative and/or complementary possibilities have also been discussed (Linley et al. 2019). Fortunately, immunogenicity data have indicated a numerically higher immune response of PCV15 against serotype 3 compared to PCV13 (Platt et al. 2020). Fortunately, the majority of the clinical isolates of serotype 3 belong to GPSC12 (highly frequent or dominant) (CC180; PMEN31) and do not appear to be closely related to GPSC83 (intermediate frequency). Thus, in the genome dataset of 13,454 isolates analyzed previously (Gladstone et al. 2019), 205 (1.5%) belong to GPSC12 (all of them of serotype 3) whereas only 23 isolates (0.2%) form part of the GPSC83 lineage (all of them of serotype 3 as well). In between these data, GPSC51 (intermediate frequency) is also composed only by serotype 3 isolates (*n* = 94), most of them of CC458. Interestingly, only two strains of GPSC83 were isolated from carriers: CAARWM01 and CAAQYD01; this is in agreement with the well-known characteristic of serotype 3 pneumococci that have only a limited capacity to form biofilms (Domenech et al. 2009, 2012).

In contrast with the case of GadA_Spn_, *S. pneumoniae* isolates of 20 different serotypes contain a *gadB*_Spn_ gene (Table 1). Although the majority of them are included in the new PCV20—which includes oligosaccharides of the serotypes 1, 3, 4, 5, 6A, 6B, 7F, 8, 9 V, 10A, 11A, 12F, 14, 15B, 18C, 19A, 19F, 22F, 23F, and 33F—there are pieces of evidence indicating that, in addition to serotype 3, the vaccine type efficacy against serotypes 19A and 19F has been suboptimal, at least in AOM prevention (Pichichero et al. 2023). Furthermore, oligosaccharides of serotypes 38, 25F, or 35F are included in neither pneumococcal vaccine. Moreover, the currently licensed vaccines, which target the capsular polysaccharide (the main virulence factor of *S. pneumoniae*), elicit no protection against nonencapsulated isolates and have likely contributed to the increased carriage of these kinds of pneumococcal isolates (Keller et al. 2016; Bradshaw and McDaniel 2019). In any case, however, whether nasopharyngeal colonization by *S. pneumoniae* and/or IPD might be required to trigger T1DM is completely unknown.

The bacterial genome is shaped by homologous recombination and horizontal gene transfer; gene acquisition, loss and replacement often lead to the emergence of novel pathogenic strains and it may represent a new challenge for public health. Often, this can be attributed to the movement of MGEs. Phages may have a number of different effects on the bacterial host cell, including loss of competence, changes in fitness, or bringing in virulence cargo genes. In a similar way, ICEs and other MGEs can also carry cargo genes that confer novel phenotypes to their new host cell, such as antibiotic resistance. In the present study, we have shown that *gadA*_Spn_-like genes appear to be circulated among diverse bacterial species with the participation of a prophage(s), whereas *gadB*_Spn_-like genes may be dispersed—mainly within pneumococcal strains—through the contribution of an ICE. The majority of the prophages are lost in pneumococci but the cargo genes—*gadA*_Spn_ and those encompassing it—have been well preserved, which suggests that they are advantageous for the bacterium. A similar situation was observed in pneumococcal genomes harboring *gadB*_Spn_ although, in this case, the putative ICE could be still detectable. A recent study has analyzed more than 1000 genomes of each of three pathogenic *Streptococcus* species revealing that ICE-associated cargo genes are not infrequent and reported an average of *ca.* 39, 16, and 51 cargo genes per genome in *S. agalactiae, S*. *pyogenes*, and *S. suis*, respectively. Likewise, a noticeable number of genes appear to correspond to phage-associated cargo genes in the same species (Lee and Andam 2022).

The glutamate-dependent system is associated with acid resistance in many bacteria including lactic acid bacteria (LAB) (De Biase and Pennacchietti 2012; Feehily and Karatzas 2013; Papadimitriou et al. 2016). The cytoplasmic pH increases due to the removal of H^+^ ions (see above). Concomitantly, the extracellular pH increases slightly due to the exchange between glutamate and the more alkaline GABA. Glutamate import and GABA export generally occur in bacteria simultaneously via a specific glutamate/GABA antiporter, a pH-dependent member of the amino acid-polyamine-organocation (APC) superfamily of transporters (del Alamo et al. 2022). Typically, in LAB, the GABA antiporter-coding gene (*gadC*) is closely located to the *gadB* (or *gadA*) gene (encoding GAD) and a transcriptional regulator-coding gene (*gadR*); the *gadB* and *gadC* genes frequently lie next or near each other. However, the genetic organization of the GAD system shows species and even strain specificity in LAB and other bacterial species, and the *gadC* and *gadR* genes may be either absent or located elsewhere in the chromosome (Gu et al. 2021). Generally, LAB species contain a GABA antiporter, but *Limosilactobacillus fermentum* possesses a GAD that is not accompanied by a GABA antiporter whereas *Limosilactobacillus reuteri* has two GABA antiporters (Cui et al. 2020). An obvious GABA antiporter could be found neither in *S. pneumoniae* nor in any other of the bacterial strains studied here but it was not totally unexpected since the members of the APC superfamily share relatively low levels of sequence identity although they have a common structural fold (Krammer and Prévost 2019). In any case, it has been shown that the *gadD3* gene of *Listeria monocytogenes* also lacks an associated antiporter but the intracellular accumulation of GABA appears to represent a standard cellular response against acidic conditions (Karatzas et al. 2012). In any case, the so-called “self-destructive or suicidal tendencies” of *S. pneumoniae* are well-known (McCarty 1985), with lysis mainly caused by the triggering of LytA, the major autolytic enzyme (Ronda et al. 1987): the autolysis will allow the release of GABA and/or GadA/GadB to the milieu. Moreover, the possibility that the use of “lytic” antibiotics (e.g., β-lactams) might increase the number of incident (new) cases of T1DM is worrisome and deserves future studies. Besides, the activity of the *S. pneumoniae* F_0_F_1_ ATPase increases as the pH of the medium decreases. This enzyme hydrolyzes ATP to generate a H^+^ gradient and regulates the intracellular pH via the pumping-out of protons, and the activity of the *atp* operon is regulated at the level of transcriptional initiation (Martín-Galiano et al. 2001). It remains to be determined whether pneumococcal strains producing Gad are more acid-resistant that those *gad*-lacking counterparts.

This study did have some limitations. First, it constitutes mainly a bioinformatic analysis aimed to investigate the presence and distribution in *S. pneumoniae* of a gene product with significant similarities to the human GAD65 protein that may work like an autoantigen on T1DM start. Although this is a working hypothesis, this is based not only on sequence comparisons but also on the close relationship between the seasonality of the infections caused by *S. pneumoniae*—a prominent human pathogen of children and the elderly—and the appearance of the first step(s) of T1DM progression. Second, it remains to be tested whether reactivity exists between purified GadA_Spn_/GadB_Spn_ and sera from diabetic patients. Third, the seasonal variations and environmental effects in non-tropical regions may not be generalizable to other areas in the world with different climates and environmental exposures. In summary, our findings suggest the possibility of early interaction between children and the pneumococcus in the pathogenesis of early-onset T1DM. More research is needed to elucidate the potential MM between the pneumococcal glutamate decarboxylase and GAD65 of pancreatic β cells.

## Supplementary Information

Below is the link to the electronic supplementary material.

Supplementary file1 (PDF 1296 KB)

Supplementary file2 (XLSX 25 KB)

Supplementary file3 (XLSX 137 KB)

## Data Availability

All data generated or analyzed during this study are included in this published article.
