# Supplementary material for: Two putative glutamate decarboxylases of Streptococcus pneumoniae as possible antigens for the production of anti-GAD65 antibodies leading to type 1 diabetes mellitus
Source: Int Microbiol. 2023 May 8;26(3):675–90. doi: 10.1007/s10123-023-00364-y (PMC10165594; doi:10.1007/s10123-023-00364-y)
Supplement: Supplementary file 1 — Supplementary file1 (PDF 1296 KB) [file 10123_2023_364_MOESM1_ESM.pdf]

Electronic Supplementary Material

Two putative glutamate decarboxylases of *Streptococcus pneumoniae* as possible antigens for the production of anti-GAD65 antibodies leading to type 1 diabetes mellitus

Ernesto García

Departamento de Biotecnología Microbiana y de Plantas, Centro de Investigaciones Biológicas Margarita Salas (CSIC), Ramiro de Maeztu 9, 28040 Madrid, Spain

Email address: e.garcia@cib.csic.es

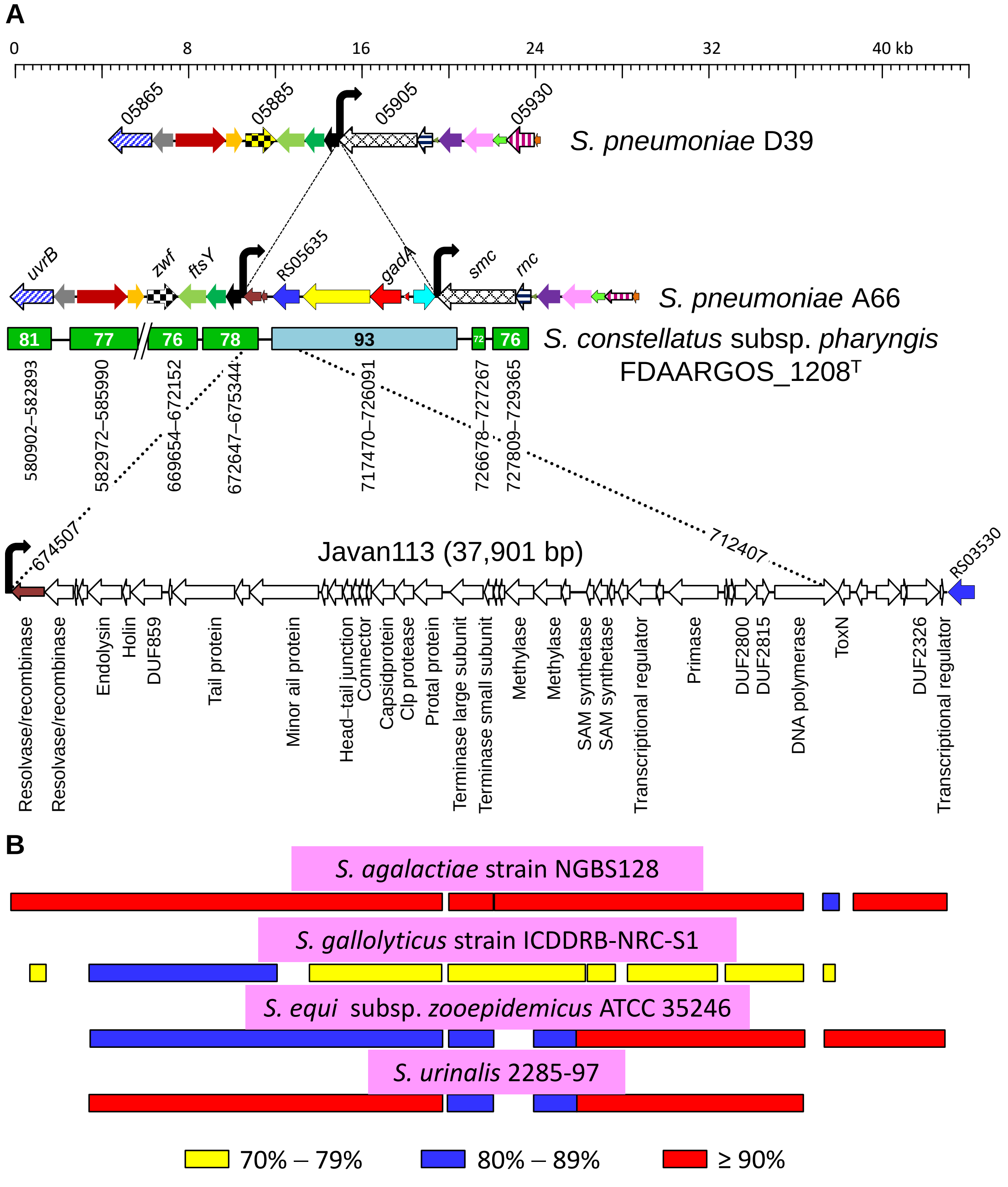

Fig. S1 (A) A comparative diagram of the chromosomal region containing a *gadA* gene in *S. pneumoniae* A66 and the type strain of *S. constellatus* subsp. *pharyngis* harboring the prophage Javan113. The percentage of nucleotide identity between those genomes is indicated in rectangles. The corresponding region of the pneumococcal D39 genome is shown for comparison. (B) Prophages of four different streptococcal species in comparison with the genome of type strain of *S. constellatus* subsp. *pharyngis* (coordinates 674507–717491 that include from the 3’ end of I6J38_RS03320 to the 3’ of I6J38_RS03530). The percentage of identical nucleotides is indicated at the bottom.

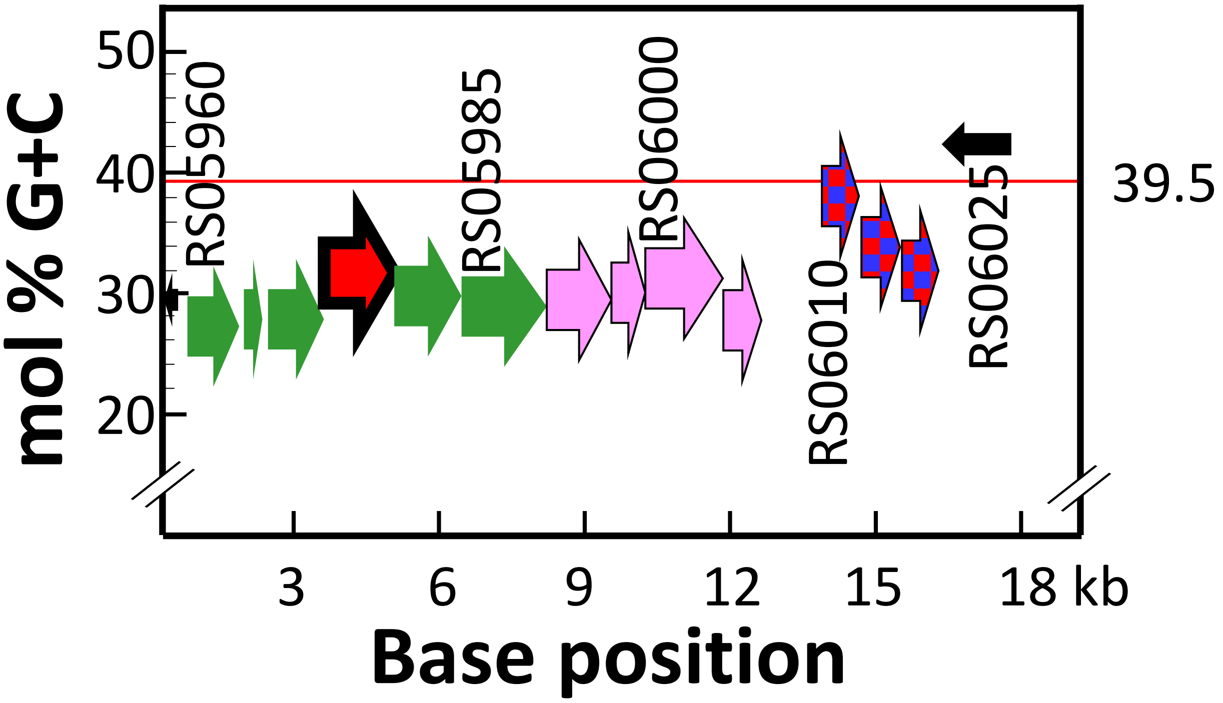

Fig. S2. Mol % G+C content of genes included in the DNA region encompassing the *gadB*$_{Spn}$ gene of *S. pneumoniae* 2245STDY6178826 (NZ_LR216061). Some genes have been identified by letters and numbers (locus tag: E0F38_RS).The G+C content of the chromosome is indicated by a red line and at the right of the figure. The color code of genes is the same of Fig. 3. The mol % G+C content was calculated for each gene.

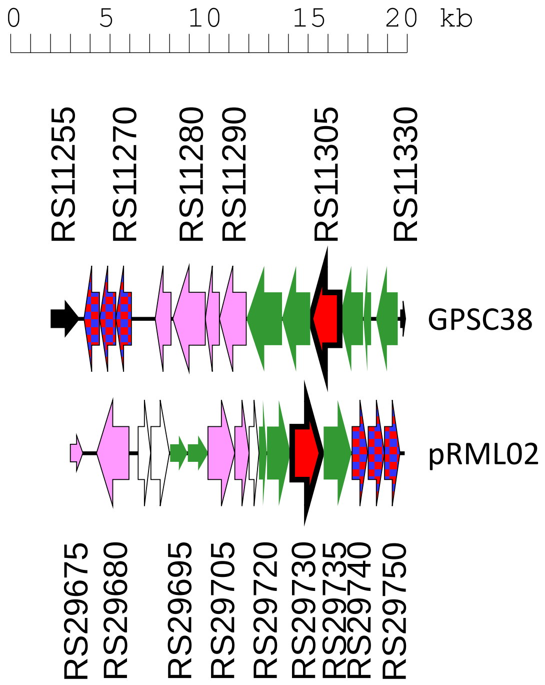

E0F14_RS	Predicted gene product	% id./simil.[a]	$Log_{10}E$	WR52_RS
11255	IS*110* family transposase (pseudo)			
11260	ABC transporter permease		ABC transporter permease	29750
11265	ABC-2 family transporter protein		ABC transporter permease	29745
11270	ATP-binding cassette domain-containing protein	40/57	−41	29740
11275	tyrosine-protein phosphatase			20675 (pseudo)
11280	methionine–tRNA ligase	63/78	≤−180	29680
11285	CatA-like O-acetyltransferase	37/60	−52	29710
11290	HP	32/50	−48	29705
11295	aminotransferase class I/II-fold pyridoxal phosphate-dependent enzyme	34/54	−31/−53	29695/29700
11300	acyl–CoA ligase	32/50	−48	29735
11305	pyridoxal-dependent decarboxylase	56/75	≤−180	29730
11310	HP	42/56	−93	29725
11315	acyl carrier protein	34/58	−10	29720
11320	HP			
11330	IS5-like element IS*1515* family transposase (pseudo)			

[a] Percentage of amino acid identity/similarity.

Fig. S3 Comparison of the genes embracing the pneumococcal *gadB*$_{Spn}$ and *gadB*$_{Bce}$ of pRML02 (NZ_CP011157; locus tag WR52_RS), a megaplasmid (141,238 bp) of *B. cereus* HN001. The color code is the same than in Fig. 2B. At the lower part of the figure, pairwise comparisons of the diverse gene products. The pneumococcal strain used for comparison was 2245STDY5605669 (GPSC38 ST310; NZ_LR216017; locus tag E0F14_RS)

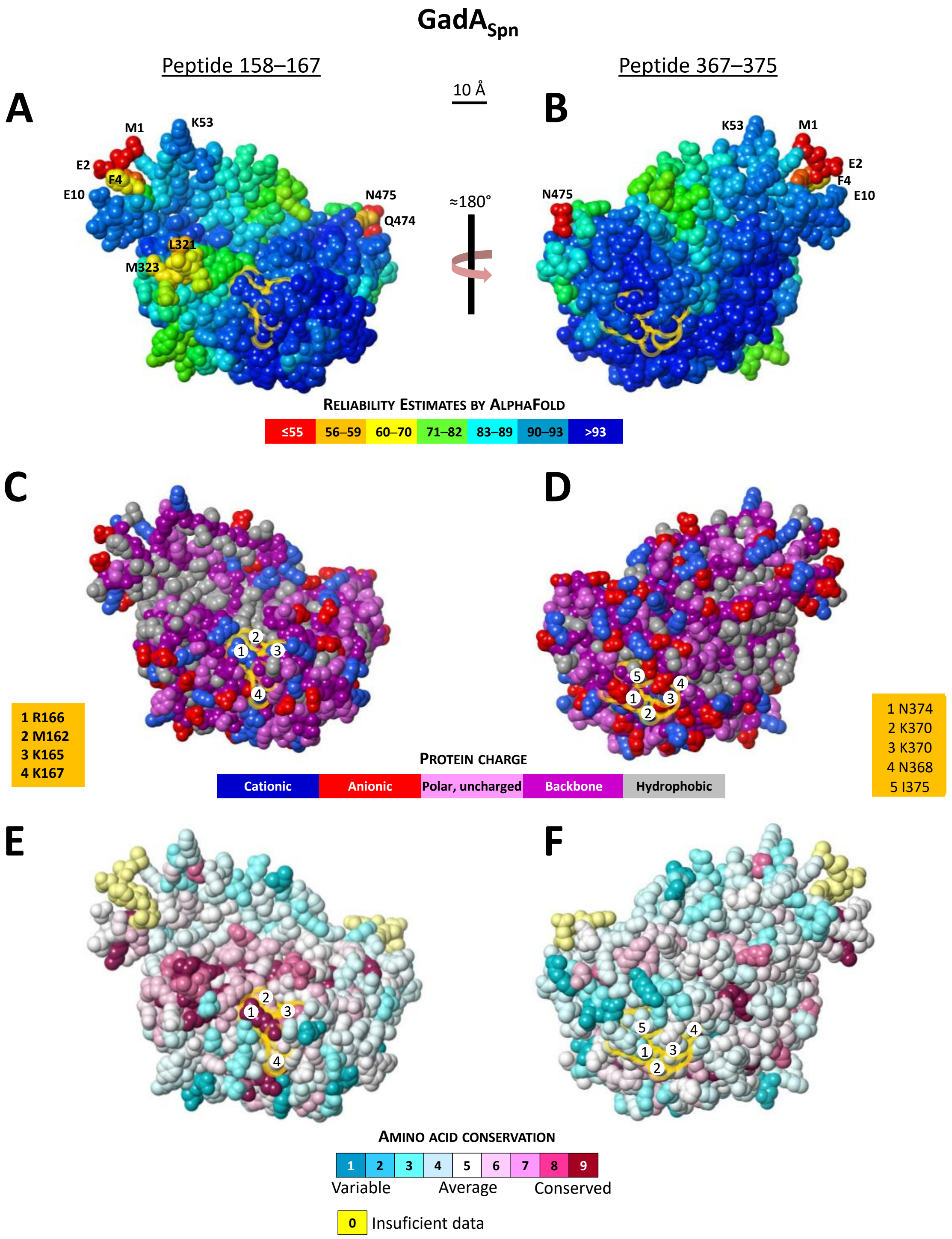

Fig. S4 Localization of two possible epitopes in the three-dimensional structure of GadA$_{Spn}$. The folding of the protein was predicted with AlphaFold and the positions of the epitopes are shown at the top and highlighted with yellow halos in each panel. (A, B) Reliability estimates. To assist orientation, the positions of several residues are included. (C, D) Charges of different amino acid residues. (E, F) Amino acid conservation calculated with ConSurf (https://consurf.tau.ac.il/) running with default parameters. Color scales are shown. In panels C to F, several surface-located amino acid residues of the predicted epitopes are indicated with numbered circles.

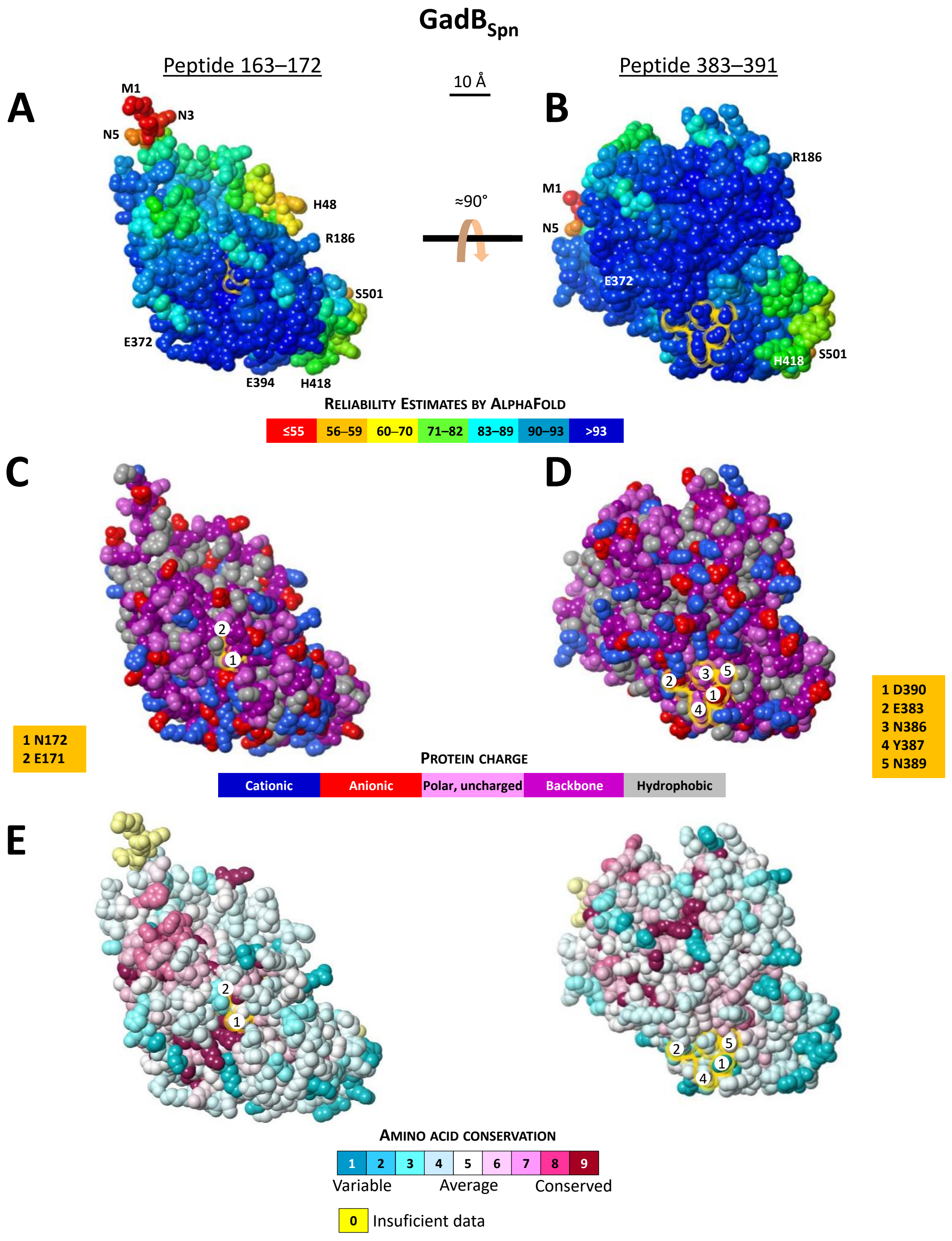

Fig. S5 Localization of two possible epitopes in the three-dimensional structure of GadB$_{Spn}$. The folding of the protein was predicted with AlphaFold and the positions of the epitopes are shown at the top and highlighted with yellow halos in each panel. (A, B) Reliability estimates. To assist orientation, the positions of several residues are included. (C, D) Charges of different amino acid residues. (E, F) Amino acid conservation calculated with ConSurf (https://consurf.tau.ac.il/) running with default parameters. Color scales are shown. In panels C to F, several surface-located amino acid residues of the predicted epitopes are indicated with numbered circles.
